# The *N* gene protects tomato plants from tomato brown rugose fruit virus infection

**DOI:** 10.1111/pbi.70237

**Published:** 2025-07-02

**Authors:** Jing Zhou, Andrea Gilliard, Jeffrey Tung, Savithramma P. Dinesh‐Kumar, Steven A. Whitham, Barbara Baker, Kai‐Shu Ling

**Affiliations:** ^1^ United States Department of Agriculture‐Agricultural Research Service U.S. Vegetable Laboratory Charleston SC USA; ^2^ United States Department of Agriculture‐Agricultural Research Service Plant Gene Expression Center Albany CA USA; ^3^ Department of Plant and Microbial Biology University of California Berkeley CA USA; ^4^ Department of Plant Biology and The Genome Center, College of Biological Sciences University of California Davis CA USA; ^5^ Department of Plant Pathology, Entomology, and Microbiology Iowa State University Ames IA USA

**Keywords:** transgenic tomato, tomato^NN^, *N* gene, tomato brown rugose fruit virus (ToBRFV), tobacco mosaic virus (TMV), tobamovirus

## Abstract

The tobamovirus tomato brown rugose fruit virus (ToBRFV) has recently emerged, causing significant damage to the tomato industry in various regions worldwide, including the US. ToBRFV evades the widely used *Tm‐2* ^*2*^ resistance gene, which encodes a nucleotide‐binding leucine‐rich repeat (NLR) class immune receptor with an N‐terminal coiled‐coil (CC) domain that confers resistance to the tomato mosaic virus (ToMV). In this study, we tested a transgenic tomato line (tomato^NN^) expressing the *Nicotiana glutinosa N* gene, which encodes an NLR with a Toll‐Interleukin 1 homology domain (TIR) at the N‐terminus, for resistance to ToBRFV. Our results demonstrate that tomato^NN^ is resistant to ToBRFV, evidenced by the necrotic local lesions observed on the inoculated leaves and the absence of symptoms on systemic leaves. This correlates with very low to non‐detectable virus levels in double antibody sandwich enzyme‐linked immunosorbent (DAS‐ELISA) and quantitative reverse transcription‐polymerase chain reaction (RT‐qPCR) assays. Furthermore, our findings reveal that tomato^NN^ is resistant to ToBRFV at 22 °C, but not at 30 °C, showing that the temperature‐sensitive nature of *N*‐mediated resistance also extends to ToBRFV resistance in tomato. These results highlight the significant potential of using tomato^NN^ to breed tomato cultivars resistant to ToBRFV, offering a new approach to managing the global pandemic caused by this emerging virus.

## Introduction

Since the discovery of the tomato brown rugose fruit virus (ToBRFV) a decade ago (Luria *et al*., 2017; Salem *et al*., 2016), this new species of tobamovirus has caused significant damage to the global tomato industry (Salem *et al*., 2023; Zhang *et al*., 2022). ToBRFV infection severely affects fruit quality and reduces yield, directly impacting marketability (Avni *et al*., 2021; Luria *et al*., 2017). The stability of ToBRFV virions facilitates dispersion through various channels, including contaminated seeds (Davino *et al*., 2020; Salem *et al*., 2022), contact with virus‐infected plant materials, soil, pollinators, tools, machinery and clothing (Chanda *et al*., 2021a,b; Klap *et al*., 2020; Levitzky *et al*., 2019). Furthermore, ToBRFV infects tomatoes that carry the *Tm‐2* and *Tm‐2* ^*2*^ genes, which encode nucleotide‐binding leucine‐rich repeat (NLR) class immune receptors with a coiled‐coil domain at the N‐terminus. It also overcomes the *Tm‐1* gene, which encodes an 80 kDa protein that binds to the replicase of the tomato mosaic virus (ToMV) (Spiegelman and Dinesh‐Kumar, 2023). Together, these factors and the virus's high transmissibility have driven its global spread.

To minimize the impact of ToBRFV, it is crucial to identify new sources of genetic resistance that can be used to breed virus‐resistant tomato cultivars. Large‐scale screenings of tomato germplasm have pinpointed ToBRFV‐resistant lines in *Solanum ochranthum, S. habrochaites, S. peruvianum*, *S. pimpinellifolium* and cultivated commercial hybrids (Jaiswal *et al*., 2024; Jewehan *et al*., 2022a,b; Zinger *et al*., 2021), as well as some tolerant lines in *S. lycopersicum*, *S. pimpinellifolium*, *S. habrochaites, S. chilense* and *S. pennellii* (Jewehan *et al*., 2022a; Kabas *et al*., 2022; Zinger *et al*., 2021). Genome‐wide association studies (GWAS), combined with quantitative trait locus (QTL) linkage mapping, have identified regions on chromosome 11 as potential candidates for ToBRFV resistance (Zinger *et al*., 2021). However, pinpointing the genes that confer resistance and incorporating them into commercial cultivars is a time‐consuming process. Unfortunately, some genotypes of ToBRFV capable of overcoming resistance in newly developed ToBRFV‐resistant cultivars have already been identified (Jewehan *et al*., 2022c; Zisi *et al*., 2024).

As one of the earliest described and most studied plant‐pathogen interactions, the relationship between the resistance gene *N* from *Nicotiana glutinosa* and tobacco mosaic virus (TMV) has long served as a classical model system for understanding host plant resistance to virus infection (Dinesh‐Kumar *et al*., 1995; Holmes, 1930, 1938; Marathe *et al*., 2002; Spiegelman and Dinesh‐Kumar, 2023). The *N* gene was isolated using transposon‐based insertional mutagenesis and encodes an NLR immune receptor with a Toll‐Interleukin 1 homology domain (TIR) at its N‐terminus (Dinesh‐Kumar *et al*., 1995; Whitham *et al*., 1994). N recognizes the TMV 126 kDa replicase, specifically the 50 kDa helicase domain (hereafter referred to as TMV‐p50), and induces a form of programmed cell death (PCD) known as the hypersensitive response (HR), which localizes the virus to the site of infection (Erickson *et al*., 1999; Padgett *et al*., 1997). *N* confers resistance to all TMV strains except for the TMV‐Ob strain (Padgett and Beachy, 1993; Tobias *et al*., 1982).

*N*‐mediated resistance to TMV is temperature‐sensitive. The *N*‐triggered HR occurs only at or below 28 °C (the permissive temperature) and is suppressed at higher temperatures, allowing unrestricted movement of the virus (Dinesh‐Kumar *et al*., 1995; Erickson *et al*., 1999; Samuel, 1931; Whitham *et al*., 1994, 1996). Interestingly, the loss of *N* function is reversible. When plants are returned to permissive temperatures, HR is restored, leading to systemic necrosis, which ultimately results in the death of the entire plant. This lethal response is due to the systemic spread of TMV at high temperatures, followed by massive cell death mediated by *N* when HR is re‐established as the temperature drops below 28 °C.

*N* isolated from *N. glutinosa* alone can confer resistance when expressed in susceptible tobacco and tomato cultivars. Transgenic *N. tabacum* cultivar Petite Havana SR1 and *N. benthamiana*, which carry the full‐length *N* genomic clone, including its native promoter and 3′ end, confer resistance to TMV (Liu *et al*., 2002; Whitham *et al*., 1994). Additionally, the TMV‐susceptible VF36 tomato line expressing full‐length genomic *N* (hereafter referred to as tomato^NN^) triggers HR in response to TMV infection and restricts the virus to the infection site, demonstrating that all essential components for *N*‐mediated resistance are preserved in tomato (Whitham *et al*., 1996). In this study, we examined whether transgenic tomato^NN^ could provide resistance to ToBRFV. Our results indicate that *N* confers resistance to ToBRFV infection in tomato^NN^. Moreover, tomato^NN^ shows resistance to ToBRFV at 22 °C but not when the temperature rises to 30 °C, indicating that temperature sensitivity is a conserved characteristic of *N*‐mediated resistance to tobamoviruses.

## Results

### The *N* gene protects tomato from ToBRFV infection

The tomato^NN^ plants (formerly designated as VTG34‐1) used in this study are transgenic tomatoes containing the genomic *N* gene, which confers resistance to TMV and ToMV (Whitham *et al*., 1996). To confirm that the tomato^NN^ plants used in this study contain the N transgene, genomic DNA prepared from the tomato^NN^ plants was tested for the presence of the *N* gene by PCR using *N*‐specific primers. We detected the expected 503 bp amplified fragment (Figure S1). In addition, Sanger sequencing confirmed the identity of the sequence. These results indicate that the tomato^NN^ plants in this study contain the *N* transgene.

**Figure 1:**
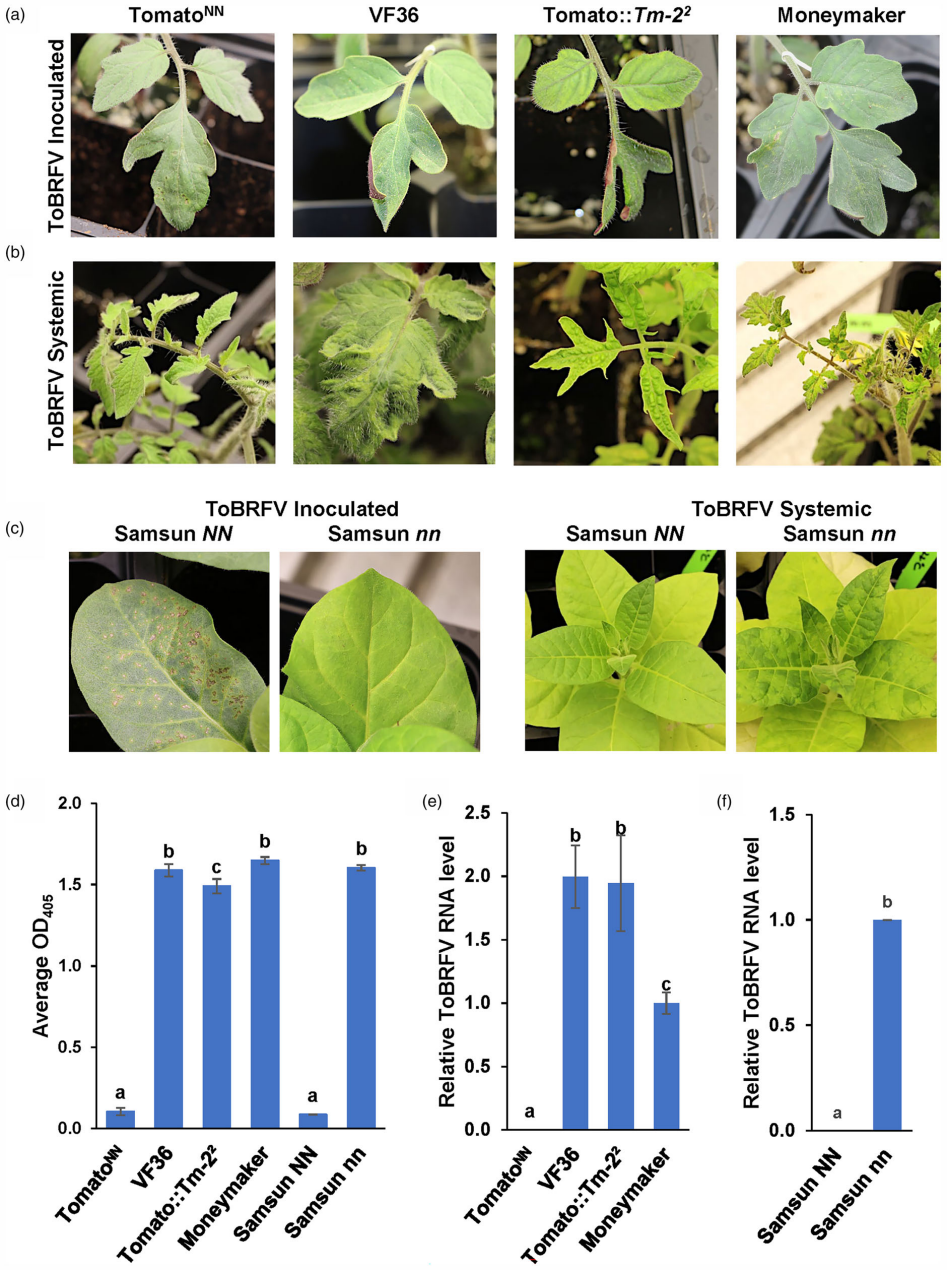
ToBRFV‐induced symptoms and virus accumulation in infected tomato and tobacco plants at 22 °C, Trial 1. (a) ToBRFV‐induced HR (hypersensitive response) local lesions appeared on all 11 inoculated tomato^NN^ leaves at 4 days post‐inoculation (4 dpi), whereas no lesions appeared on inoculated leaves of 11 VF36, Tomato::*Tm‐2* ^*2*^, or Moneymaker plants at 4 dpi. (b) Systemic leaves of 11 ToBRFV‐infected tomato^NN^ were asymptomatic at 14 dpi, whereas leaves of VF36 and Moneymaker displayed mosaic and rugose patterns, and tomato::Tm2^2^ leaves displayed rugose and shoestring growth. (c) ToBRFV‐induced HR lesions on 11 inoculated Samsun *NN* plants at 4 dpi but not on inoculated Samsun *nn* plants. Systemic leaves of ToBRFV‐infected Samsun *NN* plants remained asymptomatic at 14 dpi, whereas leaves of Samsun *nn* exhibited mosaic disease. (d) ToBRFV accumulation was measured by DAS‐ELISA assays of ToBRFV‐infected systemic tomato and tobacco leaves at 21 dpi. The Average OD_405 nm_ (Y axis) was determined as the means of measurements of 11 plants with three technical replicates for each plant in three independent experiments (Exp1‐Exp3) of each genotype (X axis). (e, f) The relative ToBRFV RNA levels (Y axes) were determined as the mean normalized RT‐qPCR Ct values (Materials and Methods) in infected systemic (e) tomato and (f) tobacco plant genotypes (Y axes) at 21 dpi, with Moneymaker set to 1 for (e) tomato and (f) Samsun *nn* set to 1 for tobacco. (d–f) Error bars indicate the SEM±, and the different letters above the bars of each graph indicate statistical differences between genotypes according to one‐way ANOVA using a Tukey HSD test, *P* ≤ 0.05 (Table 1, Trial 1, Table, Exp1‐Exp3, S1a–d).

To test whether *N* confers resistance to ToBRFV in tomato, we inoculated tomato^NN^ with ToBRFV and incubated the plants at 22 °C. As controls, we inoculated the susceptible tomato cultivars VF36 and Moneymaker, along with the tomato line LA2829, which contains the *Tm‐2* ^*2*^ resistance gene (hereafter referred to as tomato::*Tm‐2* ^2^), with ToBRFV. Additionally, we inoculated the *N. tabacum* cultivar Samsun *NN*, which contains the *N* gene, and Samsun *nn*, which lacks the *N* gene, with ToBRFV and TMV‐U1. Four days post‐inoculation (dpi), ToBRFV‐induced necrotic local lesions (HR) on inoculated leaves of tomato^NN^, but not on VF36, Moneymaker or tomato::*Tm‐2* ^*2*^ plants (Figure 1a). Consistent with recent observations (Pelletier and Moffett, 2022), we observed that HR lesions appeared on ToBRFV‐inoculated Samsun *NN* leaves at four dpi, while no lesions were observed on inoculated Samsun *nn* leaves (Figure 1c, left panel). Similarly, in accordance with previously published results (Whitham *et al*., 1994, 1996), TMV‐inoculated leaves of tomato^NN^ and Samsun *NN* exhibited HR lesions at seven dpi and two dpi, respectively (Figure S2a, top panel). These results demonstrate that ToBRFV induces an HR resistance response in tomato carrying the *N* transgene.

Next, we monitored systemic leaves of ToBRFV‐infected tomato^NN^, and VF36, Moneymaker and tomato::*Tm‐2* ^*2*^ controls for symptoms across 21 plants per genotype in three independent experiments (Exp1 to Exp3; Table 1). No symptoms were observed on the systemic leaves of tomato^NN^ plants inoculated with ToBRFV at 21 dpi (Figure 1b; Table 1). However, typical ToBRFV symptoms, including mottling and mosaic patterns, rugosity and shoestring growth, appeared on the systemic leaves of VF36, Moneymaker and tomato::*Tm‐2* ^*2*^ plants at 14 dpi (Figure 1b, Table 1). The systemic leaves of ToBRFV‐inoculated Samsun *NN* plants remained asymptomatic (Figure 1c, Table 1). In contrast, the systemic leaves of Samsun *nn* plants exhibited mosaic symptoms at 14 dpi, which intensified by 21 dpi (Figure 1c, Table 1). Consistent with prior observations (Whitham *et al*., 1994, 1996), Samsun *nn* and VF36 infected with TMV‐U1 displayed typical mosaic symptoms in their systemic leaves (Figure S2a, bottom panels). These findings indicate that ToBRFV infection of tomato^NN^ triggers HR lesions and that *N* protects plants against systemic ToBRFV infection.

**Table 1:** ToBRFV‐induced symptoms and virus accumulation in infected tomato and tobacco plants grown at 22 °C

Species		ToBRFV symptoms at 22 °C		ToBRFV detection by DAS‐ELISA, RT‐qPCR on systemic leaves^§§^				
		Trial* and Trial 2^†^ Exp1–Exp5		Trial 1*			Trial 2^†^	
	Lines/cultivars	Inoculated	Systemic	Exp1	Exp2	Exp3	Exp4	Exp5
	No. of plants	21 total	21 total	3	3	5	5	5
*Solanum lycopersicum*	Tomato^*NN*^	Local lesions^‡^	Asymptomatic^§^ ^,^ ^¶^	0/3	0/3	1/5^¶¶^	1/5^¶¶^	0/5
	VF36	Asymptomatic^††^	Mosaic, rugose^‡‡^	3/3	3/3	5/5	5/5	5/5
	Tomato::*Tm‐2* ^*2*^	Asymptomatic^††^	Rugose, shoestring^‡‡^	3/3	3/3	5/5	NT	NT
	Moneymaker	Asymptomatic^††^	Mosaic, rugose^‡‡^	3/3	3/3	5/5	NT	NT
*Nicotiana tabacum*	Samsun *NN*	Local lesions^‡^	Asymptomatic^§^	0/3	0/3	0/5	0/5	0/5
	Samsun *nn*	Asymptomatic^††^	Mosaic^‡‡^	3/3	3/3	5/5	5/5	5/5

NT, not tested.

* Trial 1: ToBRFV infection of plants in three independent experiments (Exp), Exp1–Exp3, 11 plants.

^†^ Trial 2: ToBRFV infection of plants in two independent experiments, Exp4–Exp5, 10 plants.

**Figure 2:**
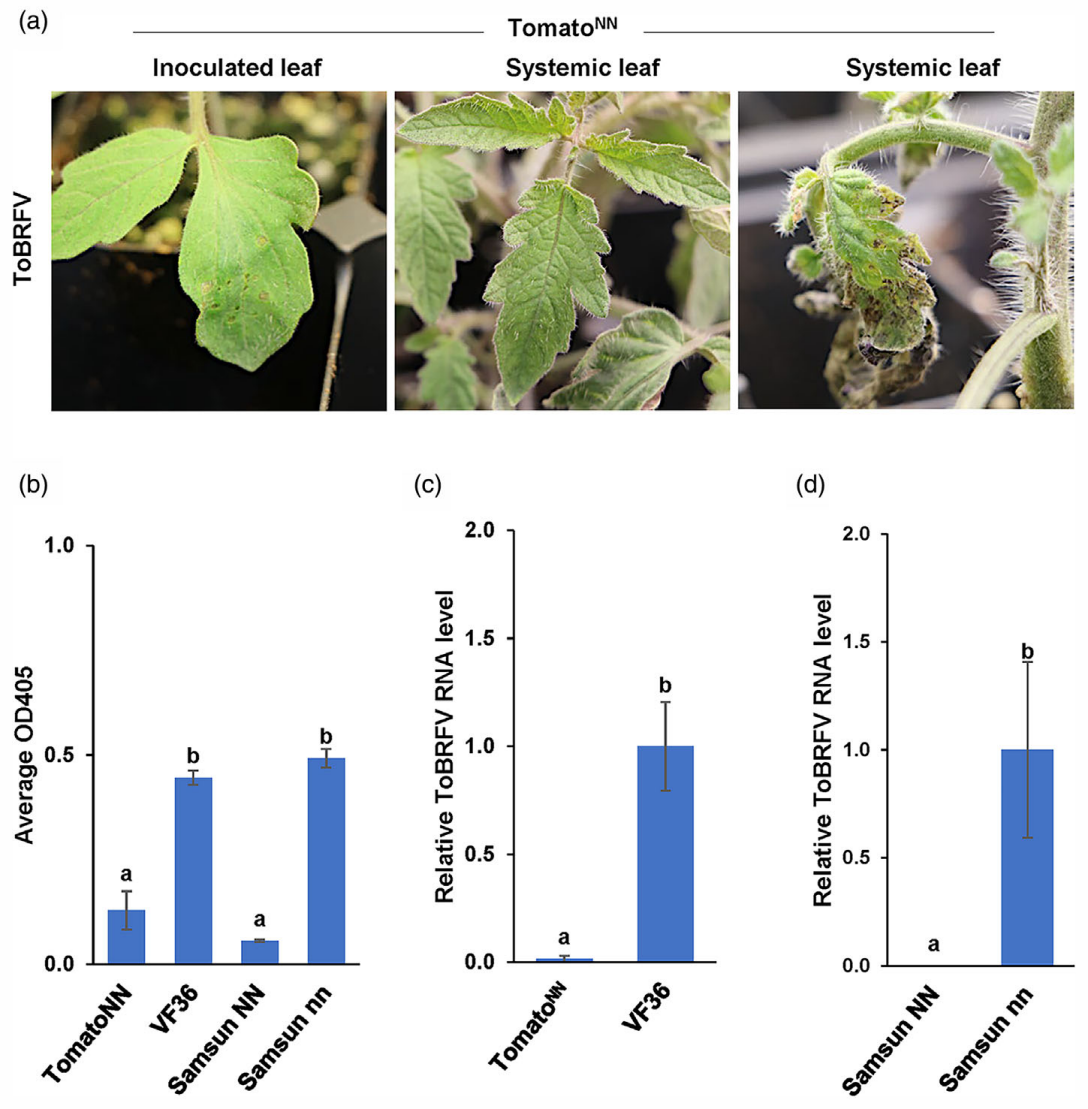
ToBRFV‐induced symptoms and virus accumulation in infected tomato plants at 22 °C, Trial 2. (a) ToBRFV‐induced HR local lesions appeared on inoculated tomato^NN^ leaves of all 10 Trial 2, Exp4‐Exp5 plants at 4 dpi (left photo, Exp4 plant 1 shown). Systemic leaves of nine out of 10 ToBRFV‐infected tomato^NN^ plants remained asymptomatic at 14 dpi (centre photo, Exp4 plant 1), whereas one of the 10 plants exhibited systemic necrosis at 14 dpi (right photo, Exp4 plant 2). (b) The Average OD_405 nm_ (Y axis) was determined as the means of measurements of 10 plants with three technical replicas for each plant in two independent experiments of genotypes shown in the X axis. (c, d) Relative ToBRFV RNA levels (Y‐axes) were determined in leaves of ToBRFV‐infected systemic (c) tomato and (d) tobacco plants as the mean of measurements of 11 plants with three technical replicas for each plant in two independent experiments (Exp4‐Exp5) of each genotype (X axes) at 21 dpi (Materials and Methods) with Moneymaker set to 1 for (c) tomato and Samsun *nn* set to 1 for (d) tobacco. (b–d) Error bars indicate the SEM±, and the different letters above the bars of each graph indicate statistical differences between genotypes according to one‐way ANOVA using a Tukey HSD test, *P* ≤ 0.05. (Table 1, Trial 2, Exp4‐Exp5, Table S1c,d).

^‡^ ToBRFV‐inoculated leaves showed local lesions on all 21 ToBRFV‐infected Tomato^NN^ at 4 dpi and 21 *N. tabacum* Samsun *NN* plants at 4 dpi (Figures 1a,c and 2a, Figure S2a).

^§^ ToBRFV‐infected systemic leaves of 20 out of 21 tomato^NN^ plants and all 21 infected systemic leaves of *N. tabacum* Samsun *NN* plants remained asymptomatic at 14 dpi, 22 °C (Figures 1b and 2a).

^¶^ ToBRFV‐infected systemic leaves of one Tomato^NN^ plant showed systemic necrosis at 14 dpi (Trial 2, Exp4 plant 2, Figure 2a).

^††^ ToBRFV‐inoculated leaves of 21 VF36, Tomato::*Tm‐2* ^2^, Moneymaker and Samsun *nn* plants remained asymptomatic at 21 dpi 22 °C (Figures 1a and 2a).

^‡‡^ ToBRFV‐infected systemic leaves of 21 VF36, Tomato::*Tm2* ^2^, Moneymaker and Samsun *nn* plants displayed ToBRFV disease symptoms at 21 dpi, 22 °C (Figure 1a).

^§§^ DAS‐ELISA and RT‐qPCR detected ToBRFV virus in systemic leaves of infected tomato and tobacco plants (Table S1a–d).

^¶¶^ DAS‐ELISA and RT‐qPCR detection of ToBRFV in systemic leaves of one tomato^NN^ plant in Trial 1 and one plant in Trial 2 (Trial 1 Exp3 plant 5: Average OD_405 nm_ = 0.34, Average Ct = 25.6; Trial 2 Exp4 plant 2: Average OD_405 nm_ = 0.52, Average Ct = 13.7 (Table S1a–d)).

### Systemic movement of ToBRFV is blocked in the majority of ToBRFV‐infected tomato^NN^ plants

To determine whether ToBRFV is present in the asymptomatic systemic leaves of tomato^NN^ plants inoculated with ToBRFV, we conducted a double antibody sandwich enzyme‐linked immunosorbent assay (DAS‐ELISA) using systemic leaves just below the growing point of each plant. The DAS‐ELISA results showed the absence of ToBRFV in the systemic leaves in 10 out of 11 tomato^NN^ plants in Trial 1, Exp1‐Exp3 (Figure 1c; Table 1). One tomato^NN^ plant, out of 5 plants, Exp3 plant 5, showed a slightly elevated DAS‐ELISA reading (OD_405_ = 0.34) compared to ToBRFV‐susceptible tomato plants (see below Average OD_405_ = 1.57) (Table 1, Table S1a). ToBRFV was detected in the systemic leaves of 11 plants of VF36, tomato::*Tm‐2* ^2^ and Moneymaker in Exp1‐Exp3 with an Average OD_405nm_ of 1.57 (Figure 1d; Table 1; Table S1a). ToBRFV was absent in systemic leaves of all Samsun *NN* plants of Exp1 to Exp3 compared to Samsun *nn* plants, in which all systemic leaves contained ToBRFV (Average OD405 = 1.6) (Figure 1d; Table 1; Table S1a).

Next, we conducted quantitative reverse transcription‐polymerase chain reaction (RT‐qPCR) analysis to further test for the presence of ToBRFV in systemic leaves of tomato^NN^ plants. In this assay, an absence of fluorescent signals (Ct value of 40) indicates that the target virus was undetected. Ten out of 11 tomato^NN^ and all 11 tobacco Samsun *NN* plant samples exhibited a Ct value of 40, signifying the absence of ToBRFV in systemic leaves of these plants (Table 1, Table S1b). Conversely, the Ct measurements in VF36, Moneymaker, tomato::*Tm‐2* ^2^ and Samsun *nn* plant samples indicated the presence of ToBRFV in the systemic leaves of infected plants (Ct values = 11.3–13.9, Table 1, Table S1b). The ToBRFV‐infected tomato^NN^ plant, Exp3 plant 5, with a DAS‐ELISA reading of OD_405_ = 0.34 (Table S1a), showed a Ct value of 25.6 (Table S1b), indicating a relatively lower amount of virus RNA was present in this plant compared to the levels of virus RNA in VF36 (Ct values = 12.2–13.9). As expected, no virus was detected in TMV‐U1‐infected systemic leaves of tomato^NN^ or Samsun *NN* controls, in contrast to VF36 and Samsun *nn* (Table S2b) by RT‐qPCR assays.

Since one plant, Exp3 plant 5, tested positive for ToBRFV in systemic leaves, we conducted two repeat experiments (Exp4 and Exp5) for Trial 2 (Table 1) to further evaluate tomato^NN^ for resistance to ToBRFV. Like the previous experiments (Exp1 to Exp3), all tomato^NN^ plants infected with ToBRFV in Exp4 and Exp5 displayed hyperplasia (HR) at 4–5 dpi on the inoculated leaves (Figure 2a, left panel). Systemic leaves of 1 of the 10 tomato^NN^ plants in Trial 2 showed necrosis at 14 dpi in Exp4 plant 2 (Figure 2a, Table 1), while the systemic leaves of the other nine tomato^NN^ plants remained asymptomatic (Figure 2a, center photo; Table 1). RT‐qPCR analysis indicated that ToBRFV is absent in all tomato^NN^ plants except for Exp4 plant 2, which exhibited systemic necrosis (Figure 2b; Table 1; Table S1d). Similarly, the absorbance reading for Exp4 plant 2 in the DAS‐ELISA assay was higher (OD_405_ = 0.52) than that of the other nine tomato^NN^ plants (Average OD_405_ = 0.075, Table S1c). As observed in Exp1 to Exp3, ToBRFV was not detected in the systemic leaves of Samsun*NN*, whereas ToBRFV was detected in VF36 and Samsun *nn* (Figure 2b,c; Table 1; Tables S1a,b). To determine if the systemic necrosis observed for Exp4 plant 2 resulted from lower or no expression of the *N* gene, we performed RT‐qPCR analysis for *N* expression in all 21 Exp1‐Exp5 plants. We found a similar level of *N* expression in all tomato^NN^ plants exhibiting resistance and the two plants exhibiting partial resistance (average Ct values = 18.6), compared to no *N* expression in Moneymaker plants (Ct values = 40) (Table S3a,b). Together, these results indicate that all tomato^NN^ plants express *N* and that ToBRFV activates N‐mediated HR, which effectively restricts the systemic spread of ToBRFV in most (~90%) of the tomato^NN^ plants at 22 °C.

### N‐mediated ToBRFV resistance is compromised in tomato^NN^ at 30 °C

**Figure 3:**
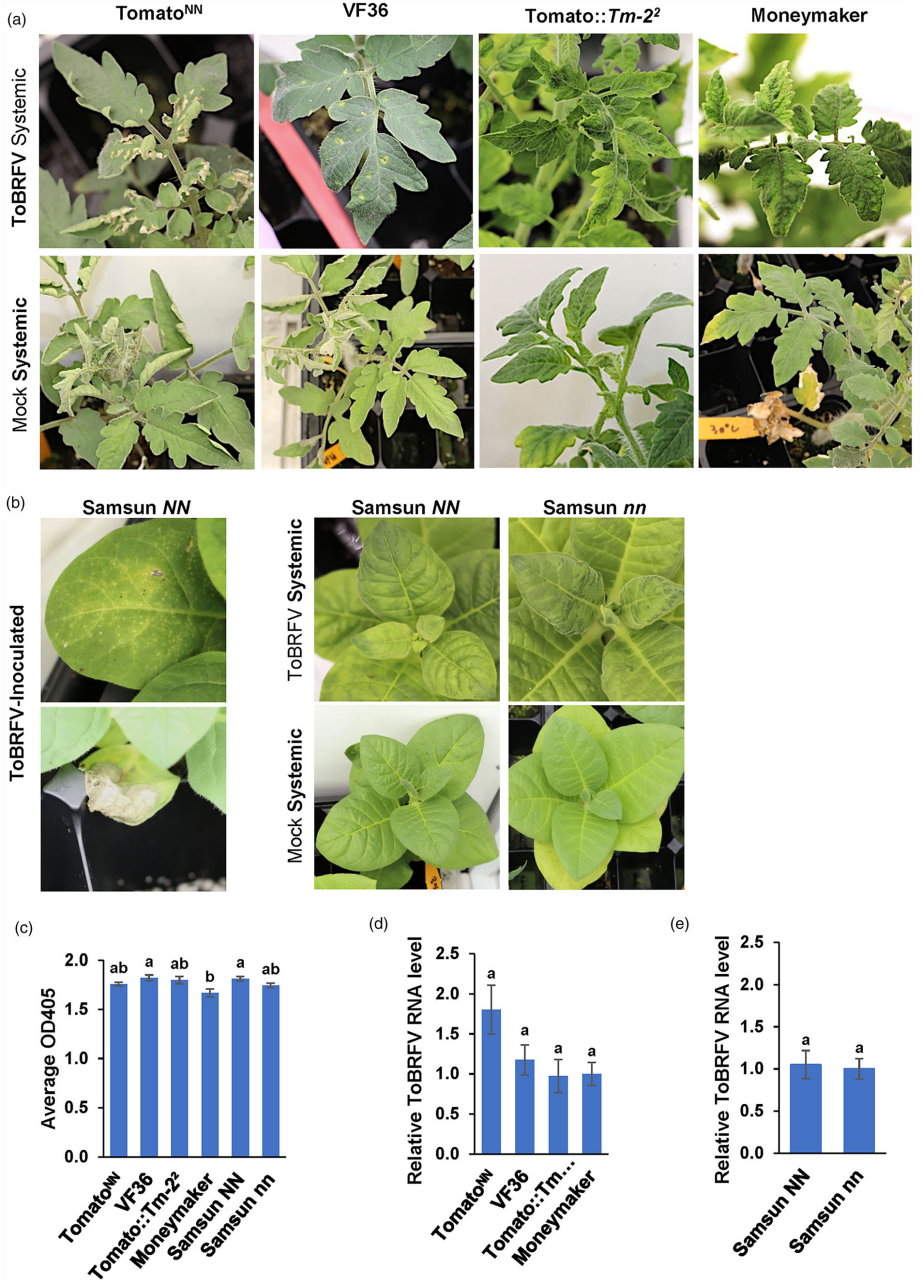
ToBRFV‐induced symptoms and virus accumulation in infected tomato and tobacco plants at 30 °C. (a) ToBRFV‐infected systemic leaves of Tomato^NN^ and VF36 plants showed scorched lesions and chlorosis at 14 dpi (upper panel). In contrast, mosaic and mottling patterns appeared on systemic leaves of tomato::*Tm‐2* ^*2*^ and Moneymaker plants at 14 dpi at 30 °C (upper panel) (Table 2). No symptoms were observed on systemic leaves of mock‐inoculated Tomato^NN^, VF36, Tomato::*Tm‐2* ^*2*^ or Moneymaker plants at 21 dpi (lower panel). (b) ToBRFV‐inoculated leaves of Samsun *NN* plants showed HR lesions and necrosis at four dpi, whereas mosaic and mottling patterns appeared on systemic leaves of Samsun *NN* and Samsun *nn* at 14 dpi. No symptoms were observed on mock‐infected systemic leaves of Samsun *NN* or Samsun *nn* plants at 21 dpi. (c) Average OD_405nm_ (Y axis) of Tomato^NN^, VF36, Tomato::*Tm‐2* ^*2*^, Moneymaker, Samsun *NN* and Samsun *nn* (X axis) determined as the mean measured for 11 plants, with three technical replicates each, in three independent experiments for each genotype. (d) The relative ToBRFV RNA levels in Tomato^NN^, VF36, *Tm‐2* ^*2*^ and Moneymaker and (e) Samsun *NN* and Samsun *nn* systemically infected leaves were determined as the mean normalized RT‐qPCR Ct values of 11 plants, with three technical replicates each, in three independent experiments for each genotype at 21 dpi, 30 °C. Error bars indicate the SEM±, and the different letters above the bars of each graph indicate statistical differences between genotypes according to one‐way ANOVA using a Tukey HSD test, *P* ≤ 0.05 (b–d) (Table 1, Exp4‐Exp5, Table S1c,d) (Table 2, Table S1e,f).

Since resistance to TMV in Samsun *NN* and tomato^NN^ is temperature‐sensitive (Whitham *et al*., 1994, 1996), we investigated whether temperature affects resistance to ToBRFV in tomato^NN^. We inoculated plants with ToBRFV, incubated them at 30 °C and monitored the symptoms. At 14 dpi, tomato^NN^ plants exhibited sporadic chlorosis and scorched lesions in the middle to upper canopy compared to the mock‐inoculated plants (Figure 3a; Table 2). VF36 plants also displayed sporadic chlorosis at 14 dpi compared to the mock control (Figure 3a; Table 2). In tomato::*Tm‐2* ^*2*^ and Moneymaker plants, typical ToBRFV symptoms, including mottling, mosaic patterns and rugosity, began to emerge on the systemic leaves at 21 dpi when compared to the asymptomatic mock‐inoculated plants (Figure 3a; Table 2). In tobacco, Samsun *NN* plants exhibited lesions and necrosis on ToBRFV‐inoculated leaves (Figure 3b), along with mottling and mosaic patterns on the upper, uninoculated leaves simultaneously, which intensified at 10–14 dpi (Figure 3c; Table 2), suggesting systemic movement of the virus. As expected, in Samsun *nn*, mosaic patterns were noted on the upper, uninoculated leaves at 14 dpi compared to the mock control (Figure 3c). Following TMV‐U1 inoculation, mosaic patterns developed on the systemic leaves of tomato^NN^ and Samsun *NN* plants kept at 30 °C (Figure S3a). The DAS‐ELISA and RT‐qPCR analysis confirmed the presence of the virus in the systemic leaves in these experiments (Figure 3d–f and Figure S3b–d). These results indicate that ToBRFV overcomes *N*‐mediated resistance in tomato^NN^ at an elevated temperature.

**Table 2:** ToBRFV‐induced symptoms and virus accumulation in tomato and tobacco plants grown at 30 °C

Species		ToBRFV symptoms at 30 °C		ToBRFV detected in systemic leaves at 30 °C^††^		
	Lines/cultivars	Inoculated leaves	Systemic leaves	Exp1	Exp2	Exp3
	No. of plants	11	11	3	3	5
*Solanum lycopersicum*	Tomato^NN^	Asymptomatic*	Sporadic chlorosis, scorched lesions^†^	3/3	3/3	5/5
	VF36	Asymptomatic*	Sporadic chlorosis^†^	3/3	3/3	5/5
	Tomato*Tm‐2* ^*2*^	Asymptomatic*	Mosaic, mottling^‡^	3/3	3/3	5/5
	Moneymaker	Asymptomatic*	Mosaic, rugose^‡^	3/3	3/3	5/5
*Nicotiana tabacum*	Samsun *NN*	Lesions and necrosis^§^	Mosaic, mottling^¶^	3/3	3/3	5/5
	Samsun *nn*	Asymptomatic*	Mosaic, mottling^¶^	3/3	3/3	5/5

* ToBRFV‐inoculated leaves of 11 Tomato^NN^, VF26, Tomato*Tm‐2* ^2^, Moneymaker and Samsun *nn* plants were asymptomatic at four dpi at 30 °C (not shown).

^†^ ToBRFV‐infected systemic leaves of 11 Tomato^NN^ plants showed sporadic chlorosis and scorched lesions, and 11 VF36 plants showed sporadic chlorosis (Figure 3a).

^‡^ ToBRFV‐infected systemic leaves of Tomato*Tm‐2* ^*2*^ and Moneymaker plants showed disease symptoms (Figure 3a).

^§^ ToBRFV‐inoculated leaves of 11 Samsun *NN* plants showed lesions and necrosis at four dpi at 30 °C (Figure 3c).

^¶^ ToBRFV‐infected systemic leaves of 11 Samsun NN and 11 Samsun *nn* showed mosaic mottling (Figure 3d).

^††^ DAS‐ELISA and RT‐qPCR detected ToBRFV in systemic leaves of infected tomato and tobacco plants (Table S1e,f).

## Discussion

Deploying cultivars with dominant resistance (*R*) genes is the most effective strategy to combat tobamoviruses. The resistance alleles *Tm‐2* and *Tm‐2* ^2^, derived from *Solanum peruvianum*, along with the semi‐dominant gene *Tm‐1*, confer resistance to TMV and ToMV (Lanfermeijer *et al*., 2003, 2005; Meshi *et al*., 1988; Pelham, 1966; Strasser and Pfitzner, 2007). Among these genes, *Tm‐2* ^*2*^ has been critical in controlling these viruses for over half a century (Meshi *et al*., 1989; Pelham *et al*., 1970). However, the recently emerged ToBRFV breaks the *Tm‐2* ^*2*^ NLR‐mediated resistance, resulting in a lack of genetic resistance against the pathogen and further fueling the global ToBRFV epidemic (Luria *et al*., 2017; Salem *et al*., 2016, 2023; Zhang *et al*., 2022). Although screens for new sources of genetic resistance are underway, building resistance requires complex breeding strategies that are labor‐intensive and time‐consuming. The emergence of novel ToBRFV further complicates the situation, isolates that evade newly developed resistant tomato cultivars due to a single amino acid change in the virus movement protein (MP) (Jewehan *et al*., 2022c; Zisi *et al*., 2024). Given these circumstances and the fact that ToBRFV is recognized through its p50 helicase domain of replicase protein by the *N* gene in *N. tabacum*, triggering HR (Pelletier and Moffett, 2022), we revisited the *N* gene, a durable tobamovirus‐resistance gene, to evaluate its effectiveness against ToBRFV in transgenic tomato.

At 22 °C, ToBRFV‐inoculated transgenic tomato^NN^ displayed HR‐like lesions on the inoculated leaves, which restricted the virus to the inoculation site. The absence of ToBRFV in the systemic leaves of nearly all the inoculated tomato^NN^ plants, as measured by DAS‐ELISA and RT‐qPCR assays, indicated that tomato^NN^ provides resistance to ToBRFV infection. However, two outliers among the 21 tested plants had low virus levels in systemic leaves. This observation suggests that ToBRFV can occasionally spread systemically in the leaves of tomato^NN^. Using RT‐qPCR, we determined that *N* is expressed at similar levels in all tested tomato^NN^ plants, including the two plants that showed low levels of ToBRFV in systemic leaves. Our results indicate that the detection of reduced levels of ToBRFV in the outlier plants did not correlate with reduced N‐expression, suggesting that other factors may contribute to the variation of N‐mediated resistance to ToBRFV in tomato at 22 °C.

Although the timing of ToBRFV‐induced HR was observed to be the same on inoculated leaves of tomato^NN^ and Samsun NN plants at 4 dpi, we found a difference between the timing of HR induction in tomato^NN^ by ToBRFV at 4 dpi and TMV at 7 dpi. We speculate that the difference in the timing of the appearance of necrotic localized lesions induced by ToBRFV and TMV in tomato^NN^ could reflect a variation in N recognition of the p50 domains of these viruses. Differences in N recognition of the two p50 proteins could impact the timing and robustness of N activation, as well as other downstream host factors required for HR induction. Together, our findings indicate that transgenic tomato^NN^ plants are a strong source of resistance to ToBRFV infection.

Temperature is a significant environmental cue that greatly influences host‐pathogen interactions (Cheng *et al*., 2013, 2019). Resistance to the tomato leaf mould fungal pathogen *Cladosporium fulvum*, conferred by *Cf* resistance genes, is suppressed at 33 °C (de Jong *et al*., 2002). The inhibitory effects of the *Arabidopsis* proteins RPM1, RPS2 and RPS4 against the bacterial pathogen *Pseudomonas syringae* pv. tomato, which expresses AvrRpm1, AvrRpt2 and AvrRps4 effectors, respectively, are less effective at 28 °C compared to 22 °C (Wang *et al*., 2009). Furthermore, the *Mi‐1* gene in tomato, which confers resistance to root‐knot nematodes (*Meloidogyne* spp.), becomes inactive above 28 °C (Abdul‐Baki *et al*., 1996). Regarding virus‐plant interactions, *Tsw*‐mediated resistance to the tomato spotted wilt virus (TSWV) fails to function at or above 32 °C in pepper (Moury *et al*., 1998). Most resistance genes identified against potato virus Y (PVY), including *Ny*, only confer resistance below 20 °C (Makarova *et al*., 2018), while all sources of resistance to wheat streak mosaic virus (WSMV), mediated by *Wsm* genes, are sensitive to temperature with variable thresholds (Farahbakhsh *et al*., 2019). Additionally, compatible virus‐plant interactions, such as tomato plants infected by the tomato yellow leaf curl virus (TYLCV), show increased susceptibility, accumulating higher virus titers at elevated temperatures (Anfoka *et al*., 2016). It is well established that *N*‐mediated resistance to TMV infection is temperature‐sensitive, with 28 °C being the threshold above which the resistance is suppressed (Marathe *et al*., 2002; Samuel, 1931; Whitham *et al*., 1996). In this study, without further defining the threshold temperature, our results indicated that ToBRFV resistance exhibited in transgenic tomato^NN^ is also influenced by temperature changes, a property comparable to the N‐TMV interaction (Whitham *et al*., 1996). Considering that temperature sensitivity is a common characteristic of NLR‐mediated resistance to plant pathogens, it is not entirely surprising to observe such similarities between the two tobamoviruses that share the highest nucleotide sequence identity (82%) among all identified tobamoviruses. However, the molecular mechanisms underlying this thermosensitive nature are still not fully understood. The N protein with Y646K or Y646K and N648D mutations corresponding to the second LRR motif induces HR at both 22 °C and 30 °C (Zhu *et al*., 2010). The loss of temperature sensitivity seems to correlate with the nuclear localization of the N protein (Zhu *et al*., 2010). These findings imply that N itself is responsible for the temperature sensitivity and could be engineered to overcome it. In the future, it will be intriguing to test whether mutations responsible for changes in nuclear localization or other mutations in N can influence the temperature sensitivity of ToBRFV‐triggered HR and resistance.

A lack of genetic resistance to ToBRFV presents a significant challenge for the global tomato industry in combating this emerging virus. However, selecting and breeding cultivars with new resistance sources is a challenging task to accomplish quickly. Based on our findings, developing tomato cultivars that carry *N* may serve as an effective source of resistance against ToBRFV infection. Our studies may contribute to understanding how different temperature regimes can be applied for antiviral defence. Additionally, a comprehensive understanding of how the temperature sensitivity of NLR‐based resistance is regulated is necessary to generate NLRs that can operate effectively across broader temperature ranges for future agricultural production.

## Materials and methods

### Plant materials and virus source

Seeds of the tomato cultivar ‘VF36’ and its transgenic line with the *N* gene (designated as tomato^NN^, previously VTG34‐1) were obtained from Drs. Barbara Baker and Savithramma P. Dinesh‐Kumar (Whitham *et al*., 1996). To confirm the presence of the *N* gene in tomato^NN^ plants, we conducted PCR amplification using *N* gene‐specific primers (N.t_N3_785F: GATTGTTGACCAAATCTCATCC and N.t_N3_1287R: CAAATGCTTGTCTCTAGTTGTT), followed by Sanger sequencing and analysis of the amplicons. For controls, tomato LA2829 containing the *Tm‐2* ^*2*^ gene (tomato::*Tm‐2* ^2^) and tomato ‘Moneymaker’ without the *Tm‐2* ^*2*^ gene, along with tobacco cultivars *N. tabacum* Samsun *NN* (*NN*) and Samsun *nn* (*nn*), were germinated and maintained in growth chambers at a temperature of 22 °C with a light cycle of 16 h light and 8 h dark. The ToBRFV pure culture was prepared using isolate ‘CA18‐01’, initially described by Ling *et al*. (2019), through a serial passage (Chanda *et al*., 2021a, GenBank Accession No. MT002973). This culture was maintained on the tomato ‘Moneymaker’ line in an insect‐proof cage inside a greenhouse at the US Vegetable Laboratory in Charleston, South Carolina. The U1 strain of TMV (TMV‐U1), provided by Dr. Barbara Baker, was maintained on Samsun *nn* through a serial passage. A single infection of each culture containing the respective virus was confirmed before use (Chanda *et al*., 2021a; Pereda *et al*., 2000).

### Mechanical inoculation of viruses

When tomato seedlings reached the first true‐leaf stage (approximately 14 days post‐germination) and tobacco seedlings had four expanded true leaves, half of the plants were transferred to a growth chamber set at 30 °C. In contrast, the other half was maintained at 22 °C. The light cycles in both chambers were identical (16 h/8 h: day/night). After 2 days of acclimation at their respective temperatures, the plants in both chambers were mechanically inoculated with either ToBRFV or TMV‐U1 (hereafter referred to as TMV). The virus inoculum was prepared by grinding freshly collected, virus‐infected leaf tissue in phosphate‐saline buffer (pH 7.0, 140 mM NaCl, 8 mM Na_2_HPO_4_, 1.5 mM KH_2_PO_4_, 2.7 mM KCl and 0.8 mM Na_2_SO_3_) at a 1:100 ratio (w/v) using a HOMEX‐6 tissue homogenizer (Bioreba AG, Reinach, Switzerland). This mixture was then rub‐inoculated onto carborundum‐dusted tomato or tobacco leaves with cotton swabs (Chanda *et al*., 2021a). Inoculated leaves were labelled for further analysis, and the plants were maintained in individual chambers for symptom development on both inoculated and systemic leaves. The mock‐inoculated group was established under the same conditions for each test line. The experiment was repeated three to five times with three to five plants per line in each repetition.

### Serological test using enzyme‐linked immunosorbent assay (ELISA)

The double antibody sandwich enzyme‐linked immunosorbent assay (DAS‐ELISA) was performed using either a TMV‐specific or ToBRFV‐specific polyclonal antibody, following the manufacturer's instructions (Agdia, Elkhart, IN, US). Briefly, leaf tissue extract (1:20 w/v) was prepared by grinding freshly collected leaves in individual sample bags with a HOMEX‐6 tissue homogenizer (BioReba, Reinach, Switzerland). After coating with an appropriately diluted antibody (1:200), the ELISA plate was loaded with the prepared leaf extract, incubated and thoroughly washed with phosphate‐buffered saline before adding the diluted enzyme conjugate (1:200). For each sample, the average optical density (OD_405 nm_) value was obtained by averaging the readings of three technical replicates from a SpectraMax microplate reader (Molecular Devices, San Jose, CA). An OD value at 405 nm was considered positive if it was at least three times that of the healthy control (Klap *et al*., 2020).

### Quantitative reverse transcription‐polymerase chain reaction (RT‐qPCR)

TaqMan® quantitative RT‐PCR (RT‐qPCR) assays to determine RNA expression used specific RT‐qPCR primers for ToBRFV, TMV and *N* specific primers: ToBRFV (Chanda *et al*., 2021a), TMV (TMV‐F: 5′‐AGCTCGAACTGTCGTTCAAA/TMV‐P: 5′‐FAM‐TGAGGTGTGGAAACCTTCACCACA‐TAMRA/TMV‐R: 5′‐CGGGTCTAATACCGCATTGT) and *N* (q_N.t_N_5334F: 5′‐GACCTCCGTCTTCCATCATAC/q_N.t_N_5383P: 5′‐FAM‐TCGAGGCTTCAAAGATGGAGTGCA‐TAMRA/q_N.t_N_5443R: 5′‐GAGTGTAATCCTTCAGCCACA). Briefly, each 20 μL reaction containing 400 ng of extracted RNA was prepared using the SuperScript III Platinum One‐Step RT‐qPCR kit (ThermoFisher, Waltham, MA), with the final concentrations of forward primer, probe and reverse primer set at 0.4 μM, 0.2 μM and 0.4 μM, respectively. The reactions and programmes were conducted similarly, with 300 ng of isolated RNA per reaction. For all samples of each genotype, three technical replicates were included when conducting RT‐qPCR using the AriaMX real‐time PCR system (Agilent Technologies, Santa Clara, CA). The programme was initiated at 50 °C for 15 min, followed by 95 °C for 2 min and then 40 cycles consisting of 95 °C for 15 s and 60 °C for 30 s for both ToBRFV and TMV detection assays. The mean quantification cycle value (Ct) was calculated using the 2^−ΔΔCt^ method for normalized RNA level (Livak and Schmittgen, 2011), employing either *actin* from *S. lycopersicum* (actin‐F: 5′‐GTTCCCAGGTATTGCTGATAGA/actin‐P: 5′‐HEX‐TTACTGCATTGGCTCCTAGCAGCA‐BHQ1/actin‐R: 5′‐CTGTATTTCCTCTCTGGTGGAG) or *tubA2* from *N. tabacum* (tubA2‐F: 5′‐GTGGCCACCATCAAGACTAA/tubA2‐P: 5′‐HEX‐CGTTGACTGGTGCCCTACTGGATT‐BHQ1/tubA2‐R: 5′‐GTCACCACCAGGAACAACA) as reference genes.

### Statistical analysis

The Average OD_405 nm_ or the average normalized threshold cycle (Ct) values (Relative RNA levels) were determined as the mean value of 11 samples (3 independent experiments, Exp1‐Exp3), or 10 (2 independent experiments, Exp4‐Exp5) measured in triplicate for each plant genotype in DAS‐ELISA or RT‐qPCR assays, respectively. The ±SEM was determined accordingly for each set of experiments for each genotype. The Average OD_405 nm_ values and the average normalized Ct values (relative RNA levels) underwent one‐way analysis of variance (ANOVA), with mean comparisons conducted using the Tukey HSD test at a probability level of 5% (*P* ≤ 0.05) with JMP Pro 18 (SAS Institute, Cary, NC).

## Author contributions

Conceptualization: K.‐S.L., B.B. and S.P.D.‐K.; methodology: J.Z., B.B., S.P.D.‐K., S.A.W., K.‐S.L., A.G. and J.T.; validation: J.Z., A.G. and K.‐S.L.; formal analysis: J.Z., B.B., S.P.D.‐K., S.A.W. and K.‐S.L.; investigation: J.Z., K.‐S.L., A.G. and J.T.; resources: K.‐S.L., B.B., S.P.D.‐K. and S.A.W.; data curation: J.Z., A.G. and K.‐S.L.; writing – original draft preparation: J.Z.; writing – review and editing: K.‐S.L., S.P.D.‐K., B.B., S.A.W., A.G. and J.T.; visualization: J.Z., K.‐S.L. and S.P.D.‐K.; supervision: K.‐S.L., B.B. and S.P.D.‐K.; project administration: K.‐S.L.; funding acquisition: K.‐S.L., S.P.D.‐K. and B.B. All authors have read and agreed to the published version of the manuscript.

## Funding

This research was partially funded by the USDA‐ARS National Plant Disease Recovery System (grant number: 6080‐22000‐032‐000D) and the USDA‐NIFA Crop Protection and Pest Management program, with grant number 2023‐70006‐40603 supporting KSL. Additionally, the ToBRFV work in the SPD‐K lab was supported by a USDA National Institute of Food and Agriculture (NIFA) award (grant number: GRANT13701090), and work in the BB lab was supported by USDA‐ARS National Plant Genetic Resources, Genomics and Genetic Improvement (CRIS 2030‐12210‐003‐000D).

## Supporting information


**Figure S1** Polymerase chain reaction (PCR) detection of the *N* gene. Genomic DNA of 21 ToBRFV‐inoculated tomato^NN^ plants of Trial 1 and Trial 2 (Exp1‐5) at 22 °C. An amplified DNA fragment of 503 bp was produced in all samples and sequenced to confirm *N* gene identity (Materials and Methods). (a) Detection of the *N* gene in 11 tomato^NN^ plants of Trial 1. Lanes 1–3: Exp1 plants 1–3, Lanes 4–6: Exp2 plants 1–3, Lanes 7–11: Exp3 plants 1–5. (b) Detection of the *N* gene in the DNA of 10 tomato^NN^ plants of Trial 2. Lanes 1–5: Exp4 plants 1–5; Lanes 6–10: Exp5 plants 1–5. L, DNA ladder; N, negative control (Samsun nn); P, positive control (Samsun NN).
**Figure S2** TMV‐induced symptoms and virus accumulation in infected tomato and tobacco plants at 22 °C. (a) TMV induces HR lesions that are visible in tomato^NN^ at seven dpi and in Samsun *NN* at two dpi (upper panel). Systemic mosaic patterns were observed on VF36 and Samsun *nn* at 21 dpi and 14 dpi, respectively (lower panel). (b) Average OD_405 nm_ values of DAS‐ELISA assays (Y axis) and the (c, d) relative TMV RNA levels determined as the mean normalized RT‐qPCR Ct values (Y axis) of virus‐inoculated (c) tomato plants and (d) tobacco plant genotypes (X axes, Materials and Methods). (b–d) Error bars indicate the SEM±, and the different letters above the bars of each graph indicate statistical differences between genotypes according to one‐way ANOVA using a Tukey HSD test, *P* ≤ 0.05 (Table S2a,b).
**Figure S3** TMV‐induced symptoms and virus accumulation in infected tomato and tobacco plants at 30 °C. (a) Mosaic patterns displayed on systemic leaves of TMV‐infected tomato^NN^ and VF36 14 dpi, 30 °C (upper panel). Mosaic patterns displayed on systemic leaves of TMV‐infected Samsun *NN* and Samsun *nn* at 21 dpi and 14 dpi, respectively, 30 °C (lower panel). (b) Average OD_405 nm_ values of DAS‐ELISA (b) and the relative TMV RNA levels measured as the mean normalized RT‐qPCR Ct values (c, d) of TMV‐inoculated plants (Table S3a,b).

**Table S1** (a) DAS‐ELISA Average OD_405_ ToBRFV‐infected systemic leaves of tomato and tobacco plants 21 dpi at 22 °C (Table 1, Trial 1, Exp1‐Exp3). (b) RT‐qPCR analysis of relative ToBRFV RNA levels in systemic leaves of ToBRFV‐infected tomato and tobacco plants at 22 °C (Table 1, Trial 1, Exp1‐Exp3). (c) Average OD_405_ DAS‐ELISA of ToBRFV‐infected systemic leaves of tomato and tobacco plants at 21 dpi, 22 °C (Table 1, Trial 2 Exp4‐Exp5). (d) RT‐qPCR analysis of the relative ToBRFV RNA levels in systemic leaves of infected tomato and tobacco plants at 22 °C (Figure 2, Table 1, Trial 2, Exp4‐Exp5). (e) Average OD_405_ of DAS‐ELISA assays of ToBRFV‐infected systemic leaves of tomato and tobacco plants at 30 °C (Table 2, Exp1‐Exp3). (f) RT‐qPCR analysis of relative ToBRFV RNA levels in systemic leaves of infected tomato and tobacco plants 30 °C (See Table 2, Exp1‐3).

**Table S2** (a) Average OD_405_ of DAS‐ELISA assays of TMV‐infected systemic leaves of tomato and tobacco plants 21 dpi at 22 °C (Figure S2b). (b) Relative TMV RNA levels in infected tomato and tobacco plants at 21 dpi, 22 °C (Figure S2c,d). (c) Average OD_405_ of DAS‐ELISA assays of TMV‐infected systemic leaves of tomato and tobacco plants at 30 °C at 21 dpi (Figure S3b). (d) RT‐qPCR analysis of relative TMV RNA levels in systemic leaves of infected tomato and tobacco plants at 30 °C (Figure S3c,d).

**Table S3** (a) Relative *N* expression levels in ToBRFV‐infected tomato^NN^ 21 dpi, 22 °C (Table 1, Trial 1, Exp1‐Exp3). (b) Relative expression level of *N* gene in tomato^NN^ ToBRFV‐infected plants 21 dpi 22 °C (Table 1, Trial 2, Exp 4 and Exp 5).

## Data Availability

The data that supports the findings of this study are available in the supplementary material of this article.

## References

[pbi70237-bib-0001] Abdul‐Baki, A.A. , Haroon, S.A. and Chitwood, D.J. (1996) Temperature effects on resistance to *Meloidogyne* spp. in excised tomato roots. HortScience, 31, 147–149.

[pbi70237-bib-0002] Anfoka, G. , Moshe, A. , Fridman, L. , Amrani, L. , Rotem, O. , Kolot, M. , Zeidan, M. *et al*. (2016) *Tomato yellow leaf curl virus* infection mitigates the heat stress response of plants grown at high temperatures. Sci. Rep. 6, 1–12. 26792235 10.1038/srep19715 PMC4726131

[pbi70237-bib-0003] Avni, B. , Gelbart, D. , Sufrin‐Ringwald, T. , Zinger, A. , Chen, L. , Machbash, Z. , Bekelman, I. *et al*. (2021) Tomato genetic resistance to tobamoviruses is compromised. Acta Hortic. 1316, 89–98.

[pbi70237-bib-0004] Chanda, B. , Gilliard, A. , Jaiswal, N. and Ling, K.‐S. (2021a) Comparative analysis of host range, ability to infect tomato cultivars with *Tm‐2* ^ *2* ^ gene and real‐time RT‐PCR detection of tomato brown rugose fruit virus. Plant Dis. 105, 3643–3652. 34058839 10.1094/PDIS-05-20-1070-RE

[pbi70237-bib-0005] Chanda, B. , Shamimuzzaman, M. , Gilliard, A. and Ling, K.‐S. (2021b) Effectiveness of disinfectants against the spread of tobamoviruses: *tomato brown rugose fruit virus* and *cucumber green mottle mosaic virus* . Virol. J. 18, 7. 33407624 10.1186/s12985-020-01479-8 PMC7787650

[pbi70237-bib-0006] Cheng, C. , Gao, X. , Feng, B. , Sheen, J. , Shan, L. and He, P. (2013) Plant immune response to pathogens differs with changing temperatures. Nat. Commun. 4, 1–9. 10.1038/ncomms3530 PMC3901997 24067909

[pbi70237-bib-0007] Cheng, Y.T. , Zhang, L. and He, S.Y. (2019) Plant‐microbe interactions facing environmental challenge. Cell Host Microbe, 26, 183–192. 31415751 10.1016/j.chom.2019.07.009 PMC6697056

[pbi70237-bib-0008] Davino, S. , Caruso, A.G. , Bertacca, S. , Barone, S. and Panno, S. (2020) *Tomato brown rugose fruit virus*: seed transmission rate and efficiency of different seed disinfection treatments. Plants, 9, 1615. 33233807 10.3390/plants9111615 PMC7699967

[pbi70237-bib-0009] Dinesh‐Kumar, S.P. , Whitham, S. , Choi, D. , Hehl, R. , Corr, C. and Baker, B. (1995) Transposon tagging of tobacco mosaic virus resistance gene *N*: its possible role in the TMV‐*N*‐mediated signal transduction pathway. Proc. Natl. Acad. Sci. USA, 92, 4175–4180. 7753780 10.1073/pnas.92.10.4175 PMC41906

[pbi70237-bib-0010] Erickson, F.L. , Holzberg, S. , Calderon‐Urrea, A. , Handley, V. , Axtell, M. , Corr, C. and Baker, B. (1999) The helicase domain of the TMV replicase proteins induces the N‐mediated defense response in tobacco. Plant J. 18, 67–75. 10341444 10.1046/j.1365-313x.1999.00426.x

[pbi70237-bib-0011] Farahbakhsh, F. , Hamzehzarghani, H. , Massah, A. , Tortosa, M. , Yasayee, M. and Rodriguez, V.M. (2019) Comparative metabolomics of temperature‐sensitive resistance to wheat streak mosaic virus (WSMV) in resistant and susceptible wheat cultivars. J. Plant Physiol. 237, 30–42. 31005806 10.1016/j.jplph.2019.03.011

[pbi70237-bib-0012] Holmes, F.O. (1930) Local and systemic increase of tomato mosaic virus. Am. J. Bot. 17, 789–805.

[pbi70237-bib-0013] Holmes, F.O. (1938) Inheritance of resistance to tobacco mosaic disease in tobacco. Phytopathology, 28, 553–561.

[pbi70237-bib-0014] Jaiswal, N. , Chanda, B. , Gilliard, A. , Shi, A. and Ling, K.‐S. (2024) Evaluation of tomato germplasm against tomato brown rugose fruit virus and identification of resistance in *Solanum pimpinellifolium* . Plants, 13, 581. 38475428 10.3390/plants13050581 PMC10934377

[pbi70237-bib-0015] Jewehan, A. , Salem, N. , Tóth, Z. , Salamon, P. and Szabó, Z. (2022a) Screening of Solanum (sections Lycopersicon and Juglandifolia) germplasm for reactions to the tomato brown rugose fruit virus (ToBRFV). J. Plant Dis. Prot. 129, 117–123. 10.1007/s41348-021-00535-x PMC8501354 34659580

[pbi70237-bib-0016] Jewehan, A. , Salem, N. , Tóth, Z. , Salamon, P. and Szabó, Z. (2022b) Evaluation of responses to tomato brown rugose fruit virus (ToBRFV) and selection of resistant lines in *Solanum habrochaites* and *Solanum peruvianum* germplasm. J. Gen. Plant Pathol. 88, 187–196.

[pbi70237-bib-0017] Jewehan, A. , Kiemo, F.W. , Salem, N. , Tóth, Z. , Salamon, P. and Szabó, Z. (2022c) Isolation and molecular characterization of a tomato brown rugose fruit virus mutant breaking the tobamovirus resistance found in wild Solanum species. Arch. Virol. 167, 1559–1563. 35507202 10.1007/s00705-022-05438-2 PMC9160144

[pbi70237-bib-0018] de Jong, C.F. , Takken, F.L. , Cai, X. , de Wit, P.J. and Joosten, M.H. (2002) Attenuation of Cf‐mediated defense responses at elevated temperatures correlates with a decrease in elicitor‐binding sites. Mol. Plant‐Microbe Interact. 15, 1040–1049. 12437302 10.1094/MPMI.2002.15.10.1040

[pbi70237-bib-0019] Kabas, A. , Fidan, H. , Kucukaydin, H. and Atan, H.N. (2022) Screening of wild tomato species and interspecific hybrids for resistance/tolerance to Tomato brown rugose fruit virus (ToBRFV). Chil. J. Agric. Res. 82, 189–196.

[pbi70237-bib-0020] Klap, C. , Luria, N. , Smith, E. , Bakelman, E. , Belausov, E. , Laskar, O. , Lachman, O. *et al*. (2020) The potential risk of plant‐virus disease initiation by infected tomatoes. Plants, 9, 623. 32422863 10.3390/plants9050623 PMC7285381

[pbi70237-bib-0021] Lanfermeijer, F.C. , Dijkhuis, J. , Sturre, M.J.G. , de Haan, P. and Hille, J. (2003) Cloning and characterization of the durable tomato mosaic virus resistance gene Tm‐2(2) from *Lycopersicon esculentum* . Plant Mol. Biol. 52, 1037–1049. 14558663 10.1023/a:1025434519282

[pbi70237-bib-0022] Lanfermeijer, F.C. , Warmink, J. and Hille, J. (2005) The products of the broken *Tm‐2* and the durable *Tm‐2* ^ *2* ^ resistance genes from tomato differ in four amino acids. J. Exp. Bot. 56, 2925–2933. 16172136 10.1093/jxb/eri288

[pbi70237-bib-0023] Levitzky, N. , Smith, E. , Lachman, O. , Luria, N. , Mizrahi, Y. , Bakelman, H. , Sela, N. *et al*. (2019) The bumblebee *Bombus terrestris* carries a primary inoculum of *Tomato brown rugose fruit virus*, contributing to disease spread in tomatoes. PLoS One, 14, e0210871. 30653593 10.1371/journal.pone.0210871 PMC6336271

[pbi70237-bib-0024] Ling, K.‐S. , Tian, T. , Gurung, S. , Salati, R. and Gilliard, A. (2019) First report of *Tomato brown rugose fruit virus* infecting tomato in the United States. Plant Dis. 103, 1439.

[pbi70237-bib-0025] Liu, Y. , Schiff, M. , Marathe, R. and Dinesh‐Kumar, S.P. (2002) Tobacco Rar1, EDS1 and NPR1/NIM1 like genes are required for N‐mediated resistance to tobacco mosaic virus. Plant J. 30, 415–429. 12028572 10.1046/j.1365-313x.2002.01297.x

[pbi70237-bib-0026] Livak, K.J. and Schmittgen, T.D. (2011) Analysis of relative gene expression data using real‐time quantitative PCR and the 2^−∆∆CT^ method. Methods, 25, 402–408. 10.1006/meth.2001.1262 11846609

[pbi70237-bib-0027] Luria, N. , Smith, E. , Reingold, V. , Bekelman, I. , Lapidot, M. , Levin, I. , Elad, N. *et al*. (2017) A new Israeli tobamovirus isolate infects tomato plants harboring *Tm‐2* ^ *2* ^ resistance genes. PLoS One, 12, e0170429. 28107419 10.1371/journal.pone.0170429 PMC5249172

[pbi70237-bib-0028] Makarova, S. , Makhotenko, A. , Spechenkova, N. , Love, A.J. , Kalinina, N.O. and Taliansky, M. (2018) Interactive responses of potato (*Solanum tuberosum* L.) plants to heat stress and infection with potato virus Y. Front. Microbiol. 9, 2582. 30425697 10.3389/fmicb.2018.02582 PMC6218853

[pbi70237-bib-0029] Marathe, R. , Anandalakshmi, R. , Liu, Y. and Dinesh‐Kumar, S.P. (2002) The tobacco mosaic virus resistance gene, N. Mol. Plant Pathol. 3, 167–172. 20569323 10.1046/j.1364-3703.2002.00110.x

[pbi70237-bib-0030] Meshi, T. , Motoyoshi, F. , Adachi, A. , Watanabe, Y. , Takamatsu, N. and Okada, Y. (1988) Two concomitant base substitutions in the putative replicase genes of tobacco mosaic virus confer the ability to overcome the effects of a tomato resistance gene, *Tm‐1* . EMBO J. 7, 1575–1581. 16453849 10.1002/j.1460-2075.1988.tb02982.x PMC457139

[pbi70237-bib-0031] Meshi, T. , Motoyoshi, F. , Maeda, T. , Yoshiwoka, S. , Watanabe, H. and Okada, Y. (1989) Mutations in the tobacco mosaic virus 30‐kD protein gene overcome *Tm‐2* resistance in tomato. Plant Cell, 1, 515–522. 2535549 10.1105/tpc.1.5.515 PMC159785

[pbi70237-bib-0032] Moury, B. , Selassie, K.G. , Marchoux, G. , Daubeze, A.M. and Palloix, A. (1998) High temperature effects on hypersensitive resistance to *Tomato spotted wilt tospovirus* (TSWV) in pepper (*Capsicum chinense* Jacp.). Eur. J. Plant Pathol. 104, 489–498.

[pbi70237-bib-0033] Padgett, H.S. and Beachy, R.N. (1993) Analysis of a tobacco mosaic virus strain capable of overcoming *N* gene‐mediated resistance. Plant Cell, 5, 577–586. 8518557 10.1105/tpc.5.5.577 PMC160295

[pbi70237-bib-0034] Padgett, H.S. , Watanabe, Y. and Beachy, R.N. (1997) Identification of the TMV replicase sequence that activates the N gene‐mediated hypersensitive response. Mol. Plant‐Microbe Interact. 10, 709–715.

[pbi70237-bib-0035] Pelham, J. (1966) Resistance in tomato to *Tobacco mosaic virus* . Euphytica, 15, 258–267.

[pbi70237-bib-0036] Pelham, J. , Fletcher, J.T. and Hawkins, J.H. (1970) The establishment of a new strain of tobacco mosaic virus resulting from the use of resistant varieties of tomato. Ann. Appl. Biol. 65, 293–297.

[pbi70237-bib-0037] Pelletier, A. and Moffett, P. (2022) N and N'‐mediated recognition confers resistance to tomato brown rugose fruit virus. MicroPubl. Biol. 2022. 10.17912/micropub.biology.000660 PMC9653555 36389119

[pbi70237-bib-0038] Pereda, S. , Ehrenfeld, N. , Medina, C. , Delgado, J. and Arce‐Johnson, P. (2000) Comparative analysis of TMV‐Cg and TMV‐U1 detection methods in infected *Arabidopsis thaliana* . J. Virol. Methods, 90, 135–142. 11064114 10.1016/s0166-0934(00)00230-5

[pbi70237-bib-0039] Salem, N. , Mansour, A. , Ciuffo, M. , Falk, B.W. and Turina, M. (2016) A new tobamovirus infecting tomato crops in Jordan. Arch. Virol. 161, 503–506. 26586328 10.1007/s00705-015-2677-7

[pbi70237-bib-0040] Salem, N.M. , Sulaiman, A. , Samarah, N. , Turina, M. and Vallino, M. (2022) Location of mechanical transmission of tomato brown rugose fruit virus in tomato seeds. Plant Dis. 106, 275–281. 34293918 10.1094/PDIS-11-20-2413-RE

[pbi70237-bib-0041] Salem, N.M. , Jewehan, A. , Aranda, M.A. and Fox, A. (2023) Tomato brown rugose fruit virus pandemic. Annu. Rev. Phytopathol. 61, 137–164. 37268006 10.1146/annurev-phyto-021622-120703

[pbi70237-bib-0042] Samuel, G. (1931) Some experiments on inoculating methods with plant viruses, and on local lesions. Ann. Appl. Biol. 18, 494–507.

[pbi70237-bib-0043] Spiegelman, Z. and Dinesh‐Kumar, S.P. (2023) Breaking boundaries: the perpetual interplay between tobamoviruses and plant immunity. Annu. Rev. Virol. 10, 455–476. 37254097 10.1146/annurev-virology-111821-122847

[pbi70237-bib-0044] Strasser, M. and Pfitzner, A.J.P. (2007) The double‐resistance‐breaking Tomato mosaic virus strain ToMV1–2 contains two independent single resistance‐breaking domains. Arch. Virol. 152, 903–914. 17238011 10.1007/s00705-006-0915-8

[pbi70237-bib-0045] Tobias, I. , Rast, A.T.B. and Maat, D.Z. (1982) Tobamoviruses of pepper, eggplant, and tobacco – comparative host reactions and serological relationships. Neth. J. Plant Pathol. 88, 257–268.

[pbi70237-bib-0046] Wang, Y. , Bao, Z. , Zhu, Y. and Hua, J. (2009) Analysis of temperature modulation of plant defense against biotrophic microbes. Mol. Plant‐Microbe Interact. 22, 498–506. 19348568 10.1094/MPMI-22-5-0498

[pbi70237-bib-0047] Whitham, S. , Dinesh‐Kumar, S.P. , Choi, D. , Hehl, R. , Corr, C. and Baker, B. (1994) The product of the tobacco mosaic virus resistance gene N: similarity to toll and the interleukin‐1 receptor. Cell, 78, 1101–1115. 7923359 10.1016/0092-8674(94)90283-6

[pbi70237-bib-0048] Whitham, S. , McCormick, S. and Baker, B. (1996) The *N* gene of tobacco confers resistance to tobacco mosaic virus in transgenic tomato. Proc. Natl. Acad. Sci. USA, 93, 8776–8781. 8710948 10.1073/pnas.93.16.8776 PMC38750

[pbi70237-bib-0049] Zhang, S. , Griffiths, J.S. , Marchand, G. , Bernards, M.A. and Wang, A. (2022) *Tomato brown rugose fruit virus*: an emerging and rapidly spreading plant RNA virus that threatens tomato production worldwide. Mol. Plant Pathol. 23, 1262–1277. 35598295 10.1111/mpp.13229 PMC9366064

[pbi70237-bib-0050] Zhu, Y. , Qian, W. and Hua, J. (2010) Temperature modulates plant defense responses through NB‐LRR proteins. PLoS Pathog. 6, e1000844. 20368979 10.1371/journal.ppat.1000844 PMC2848567

[pbi70237-bib-0051] Zinger, A. , Lapidot, M. , Harel, A. , Doron‐Faigenboim, A. , Gelbart, D. and Levin, I. (2021) Identification and mapping of tomato genome loci controlling tolerance and resistance to tomato brown rugose fruit virus. Plants, 10, 179. 33478073 10.3390/plants10010179 PMC7835962

[pbi70237-bib-0052] Zisi, Z. , Ghijselings, L. , Vogel, E. , Vos, C. and Matthijnssens, J. (2024) Single amino acid change in tomato brown rugose fruit virus breaks virus‐specific resistance in a new resistant tomato cultivar. Front. Plant Sci. 15, 1382862. 38774217 10.3389/fpls.2024.1382862 PMC11106371

