# Supplementary material for: The N gene protects tomato plants from tomato brown rugose fruit virus infection
Source: Plant Biotechnol J. 2025 Jul 2;23(10):4339–49. doi: 10.1111/pbi.70237 (PMC12483991; doi:10.1111/pbi.70237)
Supplement: Supplementary file 1 — Figure S1 Polymerase chain reaction (PCR) detection of the N gene. Genomic DNA of 21 ToBRFV‐inoculated tomatoNN plants of Trial 1 and Trial 2 (Exp1‐5) at 22 °C. An amplified DNA fragment of 503 bp was produced in all samples and sequenced to confirm N gene identity (Materials and Methods). (a) Detection of the N gene in 11 tomatoNN plants of Trial 1. Lanes 1–3: Exp1 plants 1–3, Lanes 4–6: Exp2 plants 1–3, Lanes 7–11: Exp3 plants 1–5. (b) Detection of the N gene in the DNA of 10 tomatoNN plants of Trial 2. Lanes 1–5: Exp4 plants 1–5; Lanes 6–10: Exp5 plants 1–5. L, DNA ladder; N, negative control (Samsun nn); P, positive control (Samsun NN). Figure S2 TMV‐induced symptoms and virus accumulation in infected tomato and tobacco plants at 22 °C. (a) TMV induces HR lesions that are visible in tomatoNN at seven dpi and in Samsun NN at two dpi (upper panel). Systemic mosaic patterns were observed on VF36 and Samsun nn at 21 dpi and 14 dpi, respectively (lower panel). (b) Average OD405 nm values of DAS‐ELISA assays (Y axis) and the (c, d) relative TMV RNA levels determined as the mean normalized RT‐qPCR Ct values (Y axis) of virus‐inoculated (c) tomato plants and (d) tobacco plant genotypes (X axes, Materials and Methods). (b–d) Error bars indicate the SEM±, and the different letters above the bars of each graph indicate statistical differences between genotypes according to one‐way ANOVA using a Tukey HSD test, P ≤ 0.05 (Table S2a,b). Figure S3 TMV‐induced symptoms and virus accumulation in infected tomato and tobacco plants at 30 °C. (a) Mosaic patterns displayed on systemic leaves of TMV‐infected tomatoNN and VF36 14 dpi, 30 °C (upper panel). Mosaic patterns displayed on systemic leaves of TMV‐infected Samsun NN and Samsun nn at 21 dpi and 14 dpi, respectively, 30 °C (lower panel). (b) Average OD405 nm values of DAS‐ELISA (b) and the relative TMV RNA levels measured as the mean normalized RT‐qPCR Ct values (c, d) of TMV‐inoculated plants (Table S3a,b). [file PBI-23-4339-s004.docx]

**The *N* gene protects tomato plants from tomato brown rugose fruit virus infection**

Jing Zhou^1^, Andrea Gilliard^1^, Jeffrey Tung^2,3^, Savithramma P. Dinesh-Kumar^4^, Steven A. Whitham^5^, Barbara Baker^2,3*^, and Kai-Shu Ling^1*^

**Supporting materials**


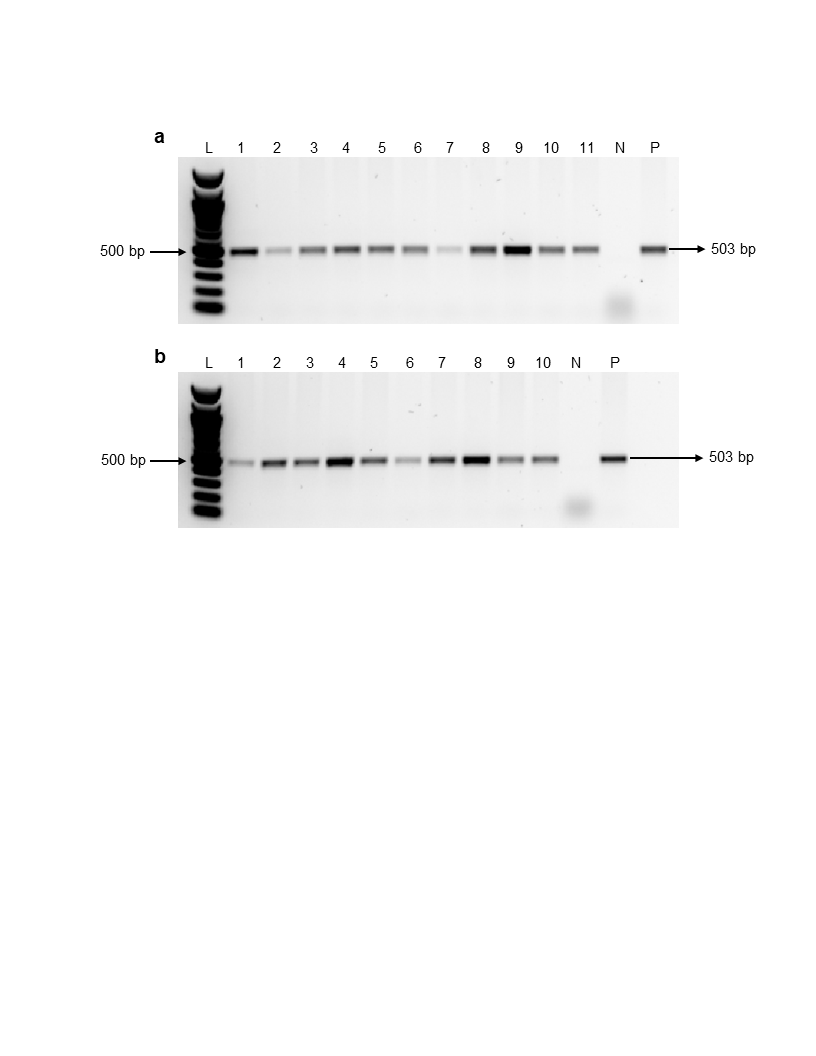


**Supplementary Figure S1.** Polymerase chain reaction (PCR) detection of the *N* gene. Genomic DNA of 21 ToBRFV-inoculated tomato^NN^ plants of Trial 1 and Trial 2 (Exp1-5) at 22°C. An amplified DNA fragment of 503 bp was produced in all samples and sequenced to confirm *N* gene identity (Materials and Methods). (**a**) Detection of the *N* gene in eleven tomato^NN^ plants of Trial 1. Lanes 1-3: Exp1 plants 1-3, Lanes 4-6: Exp2 plants 1-3, Lanes 7-11: Exp3 plants 1-5. (**b**) Detection of the *N* gene in the DNA of ten tomato^NN^ plants of Trial 2. Lanes 1-5: Exp4 plants 1-5; Lanes 6-10: Exp5 plants 1-5. L, DNA ladder; N, negative control (Samsun nn); P, positive control (Samsun NN).


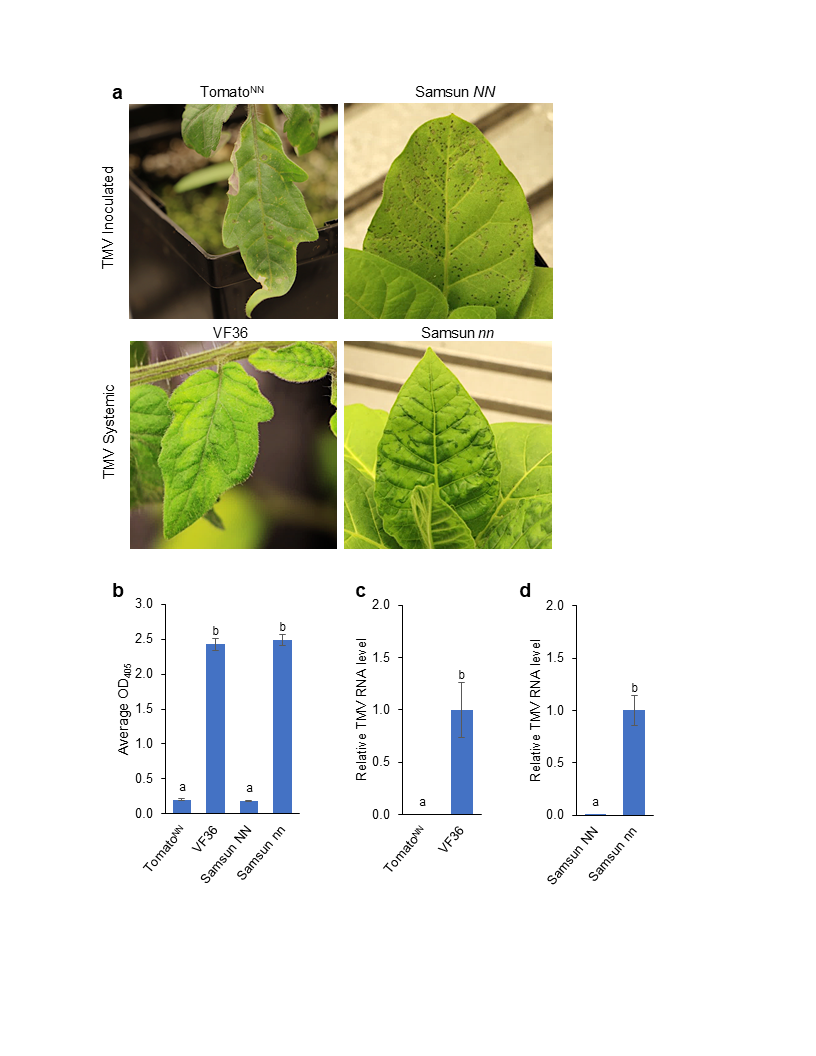


**Supplementary Figure S2**. **TMV-induced symptoms and virus accumulation in infected tomato and tobacco plants at 22°C. (a**) TMV induces HR lesions that are visible in tomato^NN^ at seven dpi and in Samsun *NN* at two dpi (upper panel). Systemic mosaic patterns were observed on VF36 and Samsun *nn* at 21 dpi and 14 dpi, respectively (lower panel). (**b**) Average OD_405nm_ values of DAS-ELISA assays (Y-axis) and the (**c, d)** relative TMV RNA levels determined as the mean normalized RT-qPCR Ct values (Y-axis) of virus-inoculated (**c**) tomato plants and (**d**) tobacco plant genotypes (X-axes, Materials and Methods). **(b, c, d)** Error bars indicate the SEM+/-, and the different letters above the bars of each graph indicate statistical differences between genotypes according to One-Way ANOVA using a Tukey HSD test, p ≤ 0.05 **(**Supplementary Tables S2a-S2b).


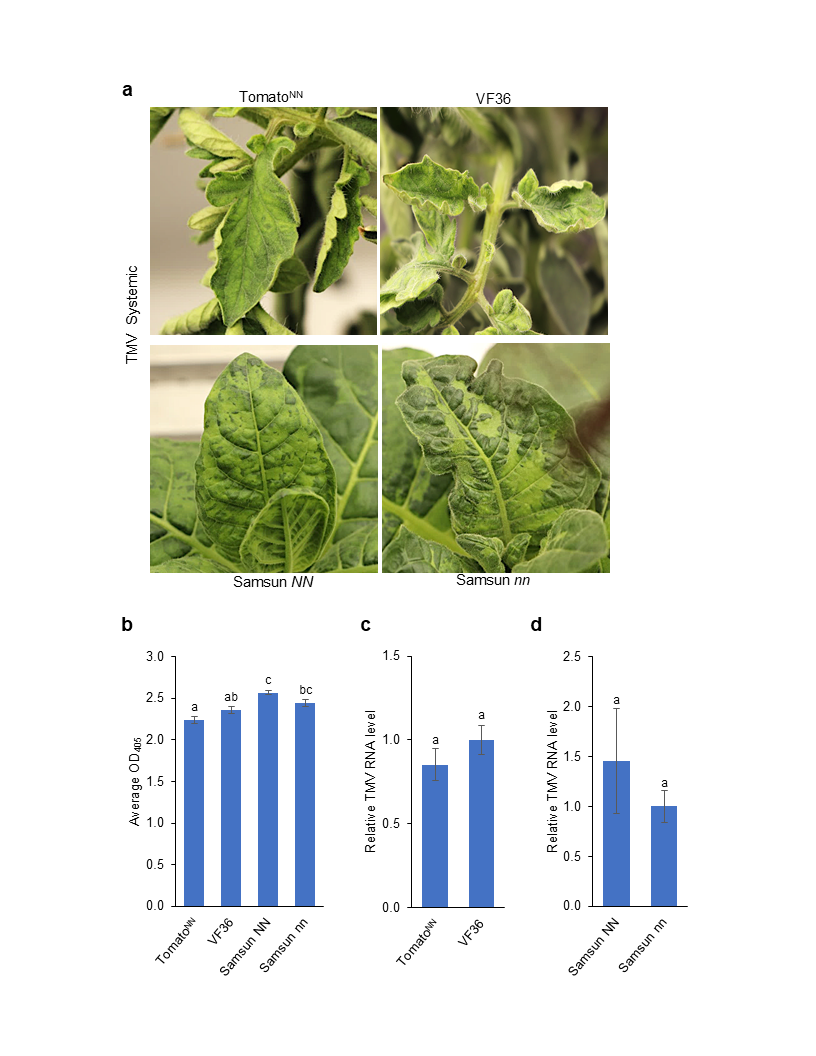


**Supplementary Figure S3.** **TMV-induced symptoms and virus accumulation in infected tomato and tobacco plants at 30°C.** (a) Mosaic patterns displayed on systemic leaves of TMV-infected tomato^NN^ and VF36 14 dpi, 30°C (upper panel). Mosaic patterns displayed on systemic leaves of TMV-infected Samsun *NN* and Samsun *nn* at 21 dpi and 14 dpi, respectively, 30ºC (lower panel). (**b)** Average OD_405nm_ values of DAS-ELISA (**b**) and the relative TMV RNA levels measured as the mean normalized RT-qPCR Ct values (**c,** **d**) of TMV-inoculated plants (Supplementary Tables S3a-S3b).
